# Co-morbid mental health conditions in people with epilepsy and association with quality of life in low- and middle-income countries: a systematic review and meta-analysis

**DOI:** 10.1186/s12955-022-02086-7

**Published:** 2023-01-20

**Authors:** Ruth Tsigebrhan, Andualem Derese, Symon M. Kariuki, Abebaw Fekadu, Girmay Medhin, Charles R. Newton, Martin J. Prince, Charlotte Hanlon

**Affiliations:** 1 Centre for Innovative Drug Development and Therapeutic Trials for Africa (CDT-Africa), College of Health Sciences, Addis Ababa University, Addis Ababa, Ethiopia; 2 Department of Psychiatry, College of Health Sciences, Addis Ababa University, Addis Ababa, Ethiopia; 3 Neuroscience Unit, KEMRI-Wellcome Trust Research Programme, Kilifi, Kenya; 4 Department of Public Health, Pwani University, Kilifi, Kenya; 5 Department of Psychiatry, WHO Collaborating Centre in Mental Health Research and Capacity-Building, School of Medicine, College of Health Sciences, Addis Ababa University, Addis Ababa, Ethiopia; 6 Global Health and Infection Department, Brighton and Sussex Medical School, Brighton, UK; 7 Aklilu-Lemma Institute of Pathobiology, Addis Ababa University, Addis Ababa, Ethiopia; 8 Department of Psychiatry, University of Oxford, Warneford Hospital, Warneford Lane, Oxford, UK; 9 Centre for Global Mental Health, Health Services and Population Research Department, Institute of Psychiatry, Psychology and Neuroscience, King’s College London, London, UK

**Keywords:** Epilepsy, Mental disorders, Neuropsychiatric comorbidity, Quality of life, Disability, Functioning, Low and middle income countries

## Abstract

**Background:**

Comorbid mental health conditions are common in people with epilepsy and have a significant negative impact on important epilepsy outcomes, although the evidence is mostly from high-income countries. This systematic review aimed to synthesise evidence on the association between comorbid mental health conditions and quality of life and functioning among people with epilepsy living in low- and middle income countries (LMICs).

**Methods:**

We searched PubMed, EMBASE, CINAHL, Global Index medicus (GID) and PsycINFO databases from their dates of inception to January 2022. Only quantiative observational studies were included. Meta-analysis was conducted for studies that reported the same kind of quality of life and functioning outcome. Cohen’s d was calculated from the mean difference in quality-of-life score between people with epilepsy who did and did not have a comorbid depression or anxiety condition. The protocol was registered with PROSPERO: CRD42020161487.

**Results:**

The search strategy identified a total of 2,101 articles, from which 33 full text articles were included. Depression was the most common comorbid mental health condition (33 studies), followed by anxiety (16 studies). Meta-analysis was conducted on 19 studies reporting quality of life measured with the same instrument. A large standardized mean effect size (ES) in quality of life score was found (pooled ES = −1.16, 95% confidence interval (CI) − 1.70, − 0.63) between those participants with comorbid depression compared to non-depressed participants. There was significant heterogeneity between studies (I^2^ = 97.6%, p < 0.001). The median ES (IQR) was − 1.20 (− 1.40, (− 0.64)). An intermediate standard effect size for anxiety on quality of life was also observed (pooled ES = −0.64, 95% CI − 1.14, − 0.13). There was only one study reporting on functioning in relation to comorbid mental health conditions.

**Conclusion:**

Comorbid depression in people with epilepsy in LMICs is associated with poor quality of life although this evidence is based on highly heterogeneous studies. These findings support calls to integrate mental health care into services for people with epilepsy in LMICs. Future studies should use prospective designs in which the change in quality of life in relation to mental health or public health interventions across time can be measured.

**Supplementary Information:**

The online version contains supplementary material available at 10.1186/s12955-022-02086-7.

## Background

Epilepsy is a serious chronic neuropsychiatric condition that affects people of all age, social class and race [1]. The prevalence of epilepsy has been estimated to be 7.6 per 1,000 population (95% CI 6.17–9.38), with a higher prevalence in low- and middle-income countries (LMICs) (8.75/1,000) compared to high income countries (HICs) (5.18/1,000) [1]. The worldwide burden of epilepsy has been observed to have decreased from 1990 to 2016, but it remains an important cause of mortality and morbidity [2]. The age standardized disability adjusted life-years (DALYs) was estimated to be 182.6 for women and 201.2 for men per 100,000 population [2]. The median standardized mortality ratio (SMR) of epilepsy ranges from 2.2 to 3.4 [3]. Both DALYs and the standardised mortality ratio (SMR) have been reported to be higher in the African region and countries with a low socio-demographic index compared to high income countries [2, 3].

Epilepsy and mental health conditions often occur with each other in a bidirectional relationship [4]. The prevalence of the most common mental health conditions in people with epilepsy (depression, anxiety disorders, psychosis, suicidality) ranges from 5 to 23%, which is 2–3 times higher than the general population [5]. Systematic reviews of studies conducted in LMICs have shown that the prevalence of co-morbid depression ranges from 24.4 to 89.9% and anxiety ranges from 1.89 to 84.2% [6]. These mental health comorbidities of epilepsy are associated with poorer treatment outcomes, higher stigma and poor quality of life according to studies carried out in HICs [7–9]. In a systematic review published in 2011, higher levels of anxiety and depression were associated with poorer quality of life [10]. However, most studies in that review were from North America and Europe (61 out of 86 studies).

Epilepsy has also been shown to be associated with increased disability (mental and physical) compared to those without epilepsy in studies done in HIC [11, 12]. In a study from Canada, disability in people with epilepsy was significantly associated with anxiety after adjusting for demographic and clinical factors [13]. In a nationally representative cross-sectional study of people with epilepsy in the USA, after adjusting for demographics and other comorbidities, only depression was significantly associated with disability [11].

Despite the high prevalence of co-morbid mental health conditions in people with epilepsy in LMICs, there is limited evidence from community-based studies especially on the impact of co-morbidity on quality of life or the related (and highly valued) patient-reported outcome of functioning. Such information is needed to advocate for increased prioritisation of co-morbidity in global programs to expand mental and neurological services to rural and underserved communities [14]. To address this gap and to identify priority areas for future research, we conducted a systematic review and meta-analysis to examine the association between comorbid mental health conditions and functioning and quality of life in people with epilepsy living in LMICs.

## Methods

The protocol for this review was registered with the international prospective register for systematic reviews (PROSPERO) CRD42020161487.

### Search strategy

The electronic databases of PubMed, EMBASE, Global Index medicus (GID), Cummulative Index to Nursing and Allied Health Literature (CINAHL) and PsycINFO were searched from their inception date until January 2022. The following key domains were searched: epilepsy, mental health conditions/mental health, quality of life, funtioning/disability and LMICs. Details of the search strategy are described in Additional file 1. There were no restrictions on language of the published articles. The reference lists of retrieved studies were manually searched for additional relevant studies. Other additional papers were identified from references of related systematic reviews and meta-analyses.

### Criteria for considering studies for this review

#### Types of studies

Any type of quantitative, observational study design (cross-sectional, case–control, cohort study) or randomised controlled trial reporting on quality of life or functioning in relation to comorbid mental health conditions in people living with epilepsy in LMICs.

### Settings

The setting of the study could be population/community-based or carried out in a health facility. The studies must have been carried out in LMICs as defined by the World Bank criteria [15].

### Population/types of participants

#### Inclusion criteria

- Epilepsy diagnosis (generalized or focal seizures) made by a clinician using the International League Against Epilepsy (ILEA) definition and classification of epilepsy, in which epilepsy is a disease of the brain defined by any of the following conditions: (a) At least two unprovoked (or reflex) seizures occurring more than 24 h apart; (b) one unprovoked (or reflex) seizure and a probability of further seizures similar to the general recurrence risk (at least 60%) after two unprovoked seizures, occurring over the next 10 years; (c) diagnosis of an epilepsy syndrome [16].
- Co-morbid mental health condition was assessed as an exposure in the study, and has been described as a medical condition that exists at the time of the diagnosis of the main condition or the index disease or later but is not the consequence of the index disease [17]. The types of mental health condition under consideration were as follows: mood disorders (major depressive disorder and bipolar disorders), anxiety disorders, somatic symptom disorders, psychotic disorders and substance use disorders. Diagnosis of comorbid mental health conditions could be by routine clinical evaluation or screening instrument or structured diagnosis by any clinician among people with epilepsy.
- Participants were 15 years of age and above so as not to exclude studies recruiting participants from adult outpatient department of hospitals which manage epilepsy from the age of 15 years.

#### Exclusion criteria

- The following mental health conditions were excluded: personality disorders, neurocognitive disorders, dissociative disorders, sexual disorders, and childhood onset psychiatric disorders e.g., attention deficit hyperactivity disorder (ADHD).
- Studies conducted in humanitarian settings.
- When participants predominantly included people with co-morbid neurodevelopmental disorders, e.g., intellectual disability or autism spectrum disorders.
- Special populations, for example, people with seizures secondary to neurological infections or substance use example alcohol withdrawal seizure.
- Functional neurological symptom disorders.
- Case reports

### Outcomes

#### Primary outcome

*Quality of life* measured using a standardised, fully structured, quantitative instrument.

*Functioning/disability* measured using a standardised, fully structured, quantitative measure.

#### Secondary outcome

*Seizure frequency*: reports of seizure frequency per month.

### Data synthesis and extraction

The identified papers were screened for eligibility criteria by two reviewers (RT and AD). Data extraction from eligible studies was done by the first author (RT) and checked by CH. All identified records with potential relevance were compiled into a database using reference management software (Endnote) [18], from where duplicates were removed. The titles and abstracts were then screened by two independent reviewers (RT and AD), to remove irrelevant studies and reports. Potentially relevant papers were retrieved, and multiple reports of the same study were linked together. All non-English articles were screened in the same fashion using Google Translate. All the steps were properly documented to construct a Preferred Reporting Items for Systematic Reviews and Meta-analysis (PRISMA) flow diagram.

A standard data extraction tool was used (Additional file 2) that included author and year, country, methods (study design, setting, study population, type of epilepsy, method for diagnosing comorbidity and type of outcome measurement tool) and findings.

### Quality and risk of bias assessment

The evaluation of the quality and risk of bias of the included studies was assessed using the Appraisal tool for cross-sectional studies (AXIS) [19] and Newcastle–Ottawa Scale (NOS) for quality appraisal for cohort and case control studies [20]. The AXIS tool has 20 questions with “yes” and “no” answers. Seven of the questions (1, 4, 10, 11, 12, 16 and 18) are about quality of reporting, seven of the questions (2, 3, 5, 8, 17, 19 and 20) are related to study design quality and six are related to the possible introduction of biases in the study (6, 7, 9, 13, 14 and 15). For this study, particular emphasis was given to quality of methodology and risk of bias (the justification of sample size, the representativeness of the participants of the target population, non-response bias). For cohort and case control studies we planned to use the Newcastle–Ottawa Scale (NOS) for quality appraisal [20].

### Data analysis

A meta-analysis of the measures of association was carried out using STATA version 17 [21]. Suitability for meta-analysis was assessed in terms of study design, measurement tools and number of studies reporting similar outcomes. Since different measures of effects were reported by different studies selected for meta-analysis, we used Cohen’s d as a desired measure of effect size with corresponding confidence interval. Cohen’s d was calculated from the mean difference in quality-of-life score between people with epilepsy who did (cases) and did not have a comorbid depression or anxiety disorder (controls or comparison group).

For those studies which did not provide the mean and standard deviation for the outcome, Cohen’s d was calculated from unstandardized and unadjusted β (regression coefficients) [22–27] or correlation coefficients [28–31] or the ANOVA t test score [32]. We used a freely available web–based calculator and effect size converter to estimate Cohen’s d with its corresponding intervals [33, 34].

Meta-analysis was carried out using a random effects model. Methodological heterogeneity (study design and risk of bias) was examined based on the above specified risk of bias tools. Statistical heterogeneity was measured using the I^2^ statistic. We conducted a sub-group analysis for those studies using clinician-based diagnosis versus those studies using a screening tool for diagnosis of comorbid mental health condition, the study setting and the income category of the study countries. The median distribution of the effect size with interquartile range (IQR) was also calculated. Meta-regression was done to see whether the subgrouping of clinician-based or screening tool diagnosis of the comorbid mental health condition or the setting (primary versus tertiary heath care) or the income category of the country (low income and lower middle income countries versus the upper middle income countries) had any significant effect on the outcome. A sensitivity analysis was conducted with the exclusion of studies rated as having a high risk of bias and poor quality. Publication bias was assessed by looking for asymmetry in funnel plots and Begg’s adjusted rank correlation test. Where there was a marked difference in the presentation of the results and where there were only two studies reporting an outcome in the same manner, only a narrative synthesis was presented.

## Results

### Description of studies

**Fig. 1:**
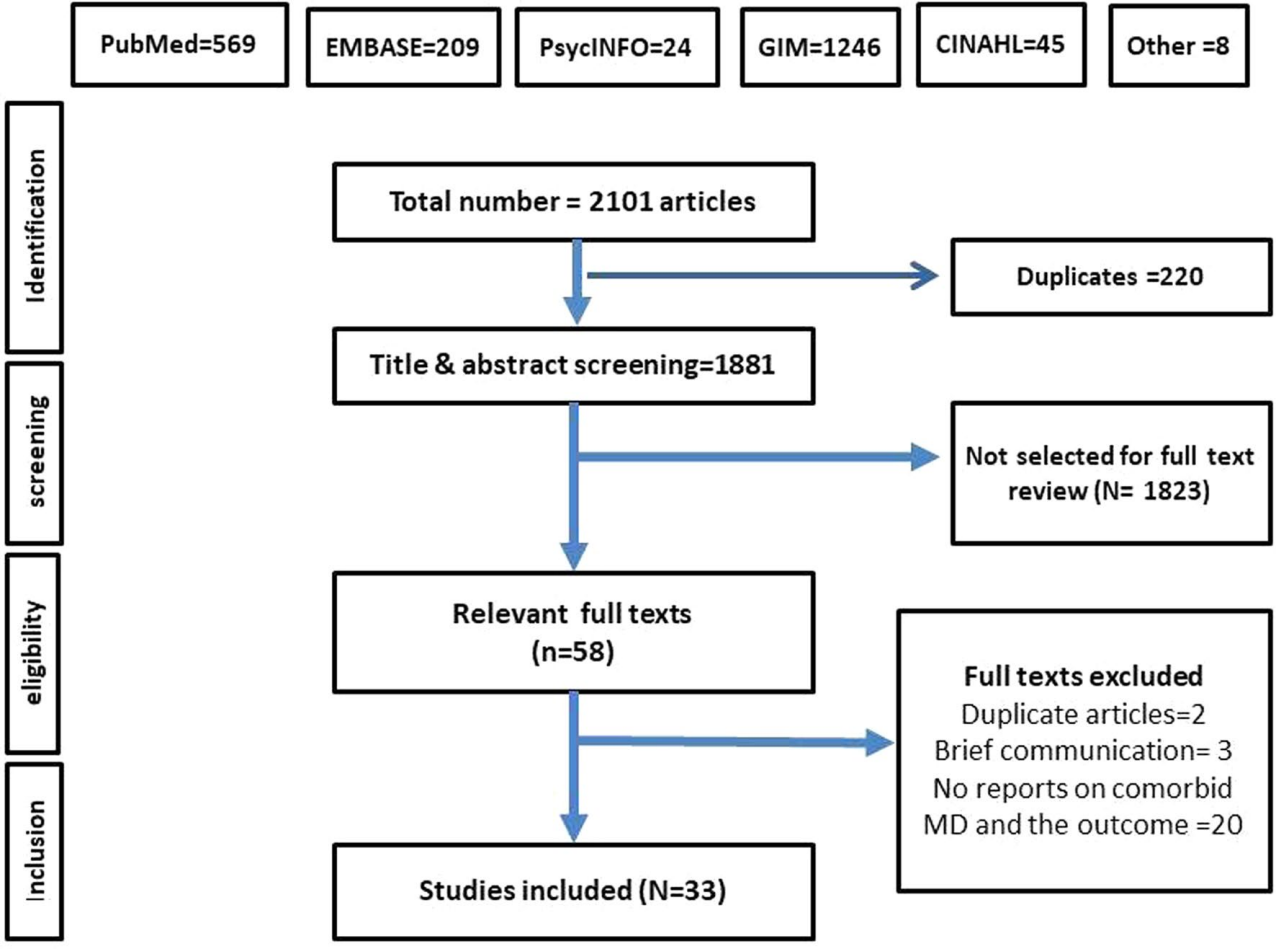
PRISMA flow chart of the selection process of included studies. ‘Other’ included articles from references of related systematic reviews and meta-analyses

The search strategy identified a total of 2,101 articles, of which 220 were duplicates and were removed. Screening of the title and the abstract led to inclusion of 58 articles for full text review. Figure 1 shows the PRISMA flow chart. After reading the full text, only 33 articles were relevant and included.

### Study design and settings

All included studies were cross-sectional, with four studies including a comparative group of participants without epilepsy [28, 35–37]. All the cross-sectional studies were able to compare the quality of life/functioning in people with epilepsy with or without comorbid mental health conditions. Twelve of the studies were conducted in sub-Saharan Africa [23–27, 32, 35, 37–41], thirteen were from Asia [29–31, 42–51], four were from Latin America [28, 52–54] and the other four studies were from Turkey and Serbia [22, 36, 55, 56]. All except seven studies (from Kenya and Ethiopia [23–27, 37, 39]) were conducted in middle-income countries. Most of the studies were carried out in tertiary health care facilities, except for two studies which were conducted at the community level [37, 46], two studies at primary [25, 51] and two other studies conducted at both primary and tertiary levels of health care [24, 26]. A summary of the included studies is presented in Table 1.

**Table 1:** Summary of included articles on the impacts of co-morbid mental health conditions in people with epilepsy

	Author’s name, year of publication	Country	Setting	Study design	Sample size	Types of epilepsy	Diagnostic method for comorbid mental disorder	Outcome measurement instrument
1	Abadiga M et al., 2019 [26]	Ethiopia	Public hospital	Cross-sectional	392	Focal and generalised	HADS	WHOQOL-BREF
2	Addis et al., 2021 [23]	Ethiopia	Tertiary hospital	Cross-sectional	370	–	HADS	QOLIE-31
3	Adebayo et al., 2014 [35]	Nigeria	Tertiary hospital	Cross-sectional	102	Focal seizure & Generalized	BDI	QOLIE-31
4	Alanis et al., 2005 [53]	Mexico	Tertiary hospital	Cross-sectional	401	Focal seizure & generalized	Clinician diagnosis of depression	QOLIE-31
5	Ayanada et al., 2020 [40]	Nigeria	Tertiary hospital;	Cross-sectional	74	–	MINI +	WHOQOL-BREF
6	Baniya et al., 2021 [49]	India	Tertiary	Cross-sectional	352	Generalised and focal	BDI-II	WHOQOL-BREF
7	Camara-Lemorroy, 2017 [28]	Mexico	Tertiary	Cross-sectional	73	Focal with or without generalization	BDI, BAI,	QOLIE-10
8	Chen et al., 2018 [29]	China	Tertiary	Cross-sectional	47	TLE	SDS, SAS	QOLIE-31
9	Ertem 2017 [36]	Turkey	Tertiary	Cross-sectional	60	MTLE JME	SCID	QOLIE-89
10	Espinosa-Jovel, 2016 [52]	Colombia	Tertiary	Cross-sectional	220	Focal seizure & generalized	NDDI-E	QOLIE-10
11	Kanchanatawan B, 2012 [42]	Thailand	Tertiary	Cross-sectional	120	–	HAMD	WHO-QOL-BREF
12	Kassie et al., 2021 [24]	Ethiopia	Primary and tertiary hospital	Cross-sectional	395	GTC & focal	PHQ-4	QOLIE-31
13	Lu Y et al., 2021 [30]	China	Tertiary Hospital	Cross-sectional	148	Generalized, focal and unclassififed	SPS	QOLIE-31
14	Meheta et al., 2014 [43]	India	Tertiary hospital	Cross-sectional	31	–	NDDI-E	QOLIE-31
15	Mesafint et al., 2020 [27]	Ethiopia	Tertiary hospital	Cross-sectional	439	–	HADS	WHO-QOL_BREF
16	Milovanović et al., 2014 [22]	Serbia	Tertiary hospital	Cross-sectional	203	–	BDI BAI	QOLIE-31
17	Mohamed et al. 2010 [44]	Malasisia	Tertiary hospital	Cross-sectional	120	Focal & Generalized	HADS MINI	QOLIE-31
18	Mosaku et al., 2006 [32]	Nigeria	Tertiary hospital	Cross-sectional	51	Focal seizure & generalized	HADS GHQ-30	QOLIE-10
19	Mwangala et al. 2018 [37]	Kenya	Community base	Cross-sectional	Cases 64	Focal seizure & generalized	MDI	The MOS 36-ItemHealth Survey
20	Olley et al., 2004 [38]	Nigeria	Tertiary	Cross-sectional	264	Focal & generalised	BDI, CCEI	Seizure control
21	Ogundare et al. 2020 [41]	Nigeeria	Tertiary hospital	Cross-sectional	270	Generalised, focal	BDI-II, MINI +	QOLIE-31
22	Phabphal K et al., 2009 [45]	Thailand	Tertiary hospital	Cross-sectional	90	–	HADS	QOLIE-31
23	Rakesh et al., 2012 [46]	India	Community	Cross-sectional	91	–	PHQ-2 GAD7	WHO-QOL-BREF
24	Senol et al., 2007 [56]	Turkey	Tertiary hospital	Cross-sectional	103	Focal seizure & generalized	BDI	QOLIE-89
25	Somayajula et al. 2015 [47]	India	Tertiary hospital	Cross-sectional	165	Myoclonic jerks with or without generalization Absence seizure	ICD-10	QOLIE-31
26	Taskiran, et al. 2019 [55]	Turkey	Tertiary hospital	Cross-sectional analysis	105	Focal seizure & generalized	BAI &BDI	QOLIE-31
27	Tedrus et al., 2013 [54]	Brasil	Tertiary hospital	Cross-sectional	132	Focal seizure & generalized	Clinician based on DSM-IV or ICD-10	QOLIE-31
28	Tegegne et al., 2014 [39]	Ethiopia	Tertiary hospital	Cross-sectional	415	–	HADS	WHOQOL-BREF
29	Tsigebrhan et al. 2020 [25]	Ethiopia	Primary health care	Cross-sectional	237	Generalised	OPCRIT	QOLIE-10
30	Wang et al., 2018 [51]	China	Primary health care	Cross-sectional	458	–	NDDI-E and GAD-7	QOLIE-31
31	Zhang H, 2021 [31]	China	Tertiary hospital	Cross-sectional	165	Generalised, focal and unclassified	NDDI-E	QOLIE-31
32	Zhao et al. 2012 [48]	China	Tertiary hospital	Cross-sectional	140	Focal seizure & generalized	HAMD-17	QOLIE-31
33	Zhong R et al. 2021 [50]	China	Tertiary hospital	Cross-sectional	221	Generalised, focal and unclassified	PHQ-9, BAI	QOLIE-31

*BAI* Beck anxiety inventory, *BDI* Beck Depression Inventory, *CCEI* the Crown Crisp Experiential Index, *DSM* Diagnostic and Statistical Manual of mental disorders, *GHQ* General Health Questionnaire, *GAD-7* Generalized anxiety disorder, *HADS* Hospital Anxiety and Depression scale, *HAMD* Hamilton depression rating scale, *ICD* International Classification of Psychiatric Disorders, *MDI* Major Depression Inventory, *MINI* Mini International neuropsychiatric interview, *MOS-36* short version of SF-36, *NDDIE* Neurological Disorders Depression Inventory for epilepsy, *OPCRIT* Operational criteria for research, *PHQ* Patient Health Questionnaire, *QOLIE* Quality Of Life for Epilepsy, *SAS* Self-rating Anxiety Scale, *SCID* Structured Clinical Interview for DSM IV, *SDS* the Self-rating Depression Scale, *SPS* social phobia scale, *WHOQOL-BREF* World Health Organization Quality of Life questionnaire

### Participants

A total of 6518 people with epilepsy (PWE) (Males = 3294, 50.5%) participated in the 33 studies. Study sample sizes ranged from 31 to 458. Eighteen of the studies included people with focal and/or generalized types of epilepsy [24, 26, 30–32, 35, 37, 38, 41, 44, 48–50, 52–56], four of them were conducted with people with focal seizures only [28, 29, 36, 47] and one article included people with generalised seizure only [25]. Clinician diagnosis of epilepsy was reported in all studies. Additional modes of investigation, e.g. electroencephalogram (EEG), were used in some of the studies. One study presented the results separately for two types of seizure disorder: mesial temporal lobe epilepsy (MTLE) and JME (Juvenile myoclonic epilepsy) [36]. Six of the reviewed studies did not report the types of epilepsy [22, 23, 39, 40, 42, 43, 45, 46, 51]. The duration of epilepsy was also reported in all studies except one [51]. In those studies which reported the mean duration of epilepsy, it ranged from 5.89 to 22 years [22, 23, 30–32, 36, 38, 41–43, 47–50, 53, 55–57].

### Assessment of comorbidities

Depression was the most commonly reported comorbid mental health condition, followed by anxiety (Table 1). The Beck Depression Inventory (BDI) was the most commonly used screening instrument for depression [22, 28, 35, 38, 41, 49, 55, 56], followed by the Hospital Anxiety and Depression Scale (HADS) [23, 26, 27, 32, 39, 44, 45]. Seven of the studies used a structured or semi-structured clinician diagnostic tool to measure comorbid mental health conditions [25, 36, 40, 41, 47, 53, 54]. Four studies reported on comorbid conditions other than depression or anxieties, including schizophrenia, sleep disorders, substance use disorders and somatoform disorders [25, 36, 47, 53]. One study measured social anxiety [30]. One study used both screening instruments and a confirmatory diagnostic tool for depression [44]. Other screening scales included the Beck Anxiety Inventory (BAI) [22, 28, 50, 55] (Table 1). Nineteen of the studies used a validated screening instrument for their respective setting and study population [27–32, 36–39, 41, 44, 46, 48, 50–52, 55, 56].

### Quality of life measures

Quality of life was the most commonly reported outcome, measured using the Quality of Life in Epilepsy scale (QOLIE)-(10, 31 and 89 item versions) in 24 of the studies, although only fifteen of the studies used a validated version of the instrument for their specific population [22, 28–31, 36, 41, 44, 48, 50–53, 55, 56]. The World Health Organization Quality of Life questionnaire (WHOQOL-BREF), a generic measure of quality of life, was used in seven studies [26, 27, 39, 40, 42, 46, 49]. One community-based study from Kenya assessed quality of life using the Medical Outcome Study-36 instrument (MOS-36) [37].

### Comorbid depression and quality of life

The mean difference in quality of life between people with epilepsy with and without depression was the most common way of reporting the association (14 out of 33 studies) [36, 37, 41, 44–49, 51, 53–56]. Two thirds of the reviewed studies presented the final result after adjusting for possible confounders [22, 24–30, 32, 35, 39–42, 44–46, 49, 50, 52, 53, 56–58]. Two studies presented the result as odds ratio [39, 40]. One study reported the association of depression with quality of life separately for women and men [50].

**Fig. 2:**
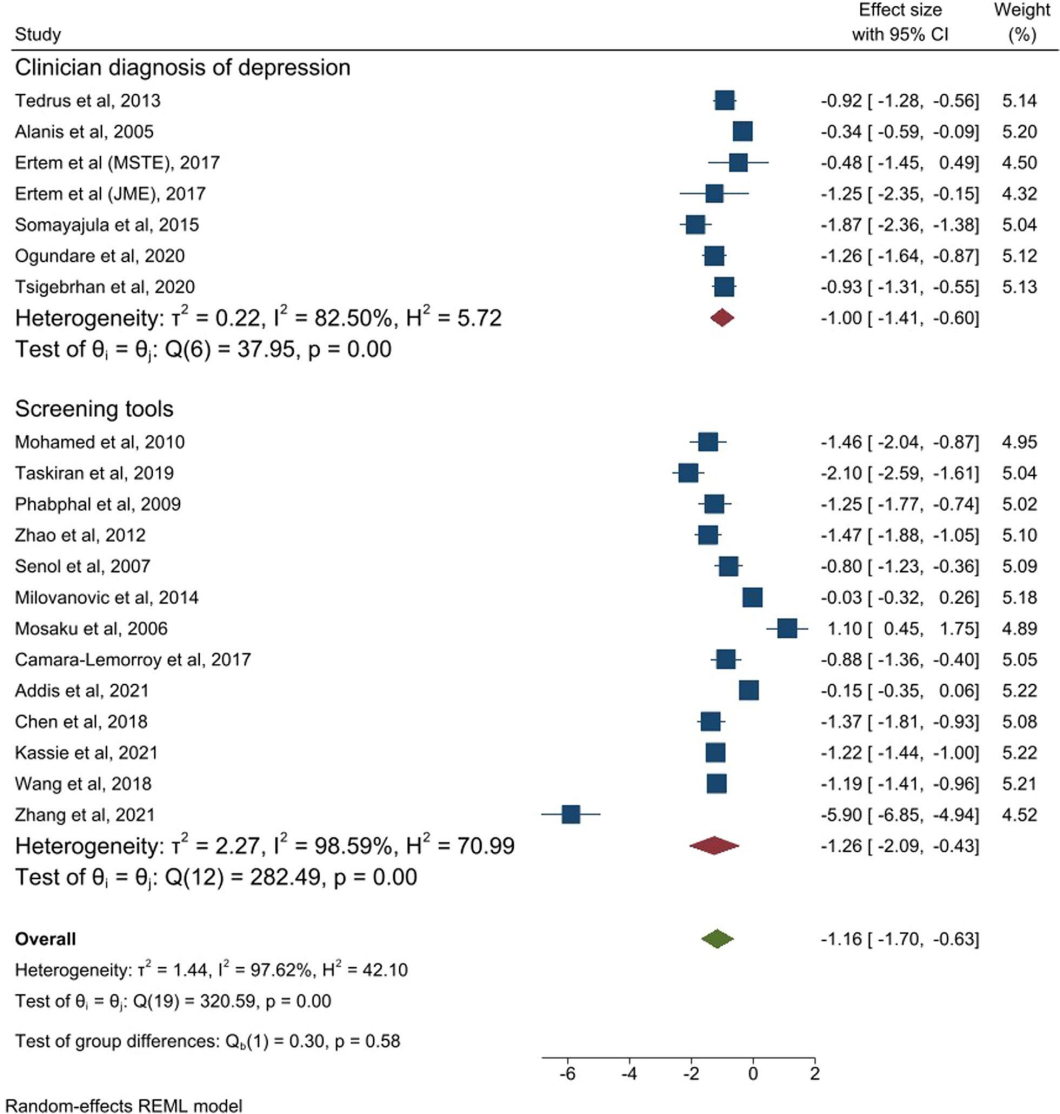
Forest plot of all the studies reporting the quality of life (QOLIE) in association of comorbid depression. *MSTE* mesial temporal lobe epilepsy, *JME* Juvenile temporal lobe epilepsy

When the nineteen studies reporting the outcome (quality of life) measured with the same instrument (QOLIE) were pooled together, a large standardized negative mean effect size (ES) was found (pooled ES = −1.16, 95% confidence interval (CI) − 1.70, (− 0.63)) between those participants with comorbid depression compared to non-depressed participants. There was significant heterogeneity between the studies (I^2^ = 97.6%, p < 0.001). The median ES (IQR) was − 1.20 (− 1.40, (− 0.64). In the sub-group analysis, those six studies which used a clinician-based diagnosis of comorbid depression had a similar standardised mean effect size (ES = -1.00, 95% CI − 1.40, (− 0.60), I^2^ = 82.5) to those that used screening tools (ES = −1.26, 95% CI − 2.60, (− 0.43), I^2^ = 98.6%) (see Fig. 2). Further sub-group analysis based on country income categories found that the standard mean effect size was intermediate for those studies done in low income and lower middle income countries but not significant (ES = −0.74, 95% CI − 1.55, 0.08) (I^2^ = 97.2%). The same ES was found between the sub-groups categorised based on the setting of the studies (see Additional file 3). Meta-regression of the ES on the diagnostic method of depression found no significant difference in ES between those studies using clinician based diagnosis and a screening tools for depression (coefficient = −0.24, 95% CI − 1.41, 0.92, p value = 0.68). There was also no significant difference in ES with meta-regression of sample size (coefficient = 0.001, 95% CI − 0.004, 0.005) or between those studies conducted in low-income versus upper middle-income countries (coefficient = −0.63, 95% CI − 1.80, 0.54) or those studies recruiting participants from primary versus tertiary health care (coefficient = −0.06, 95% CI − 1.60, 1.47).When sensitivity analysis was done by exclusion of studies with poor quality [32, 36, 54], a larger standardised mean effect size was observed (ES = −1.35, 95% CI − 1.95, (− 0.75), I^2^ = 97.9%).

**Fig. 3:**
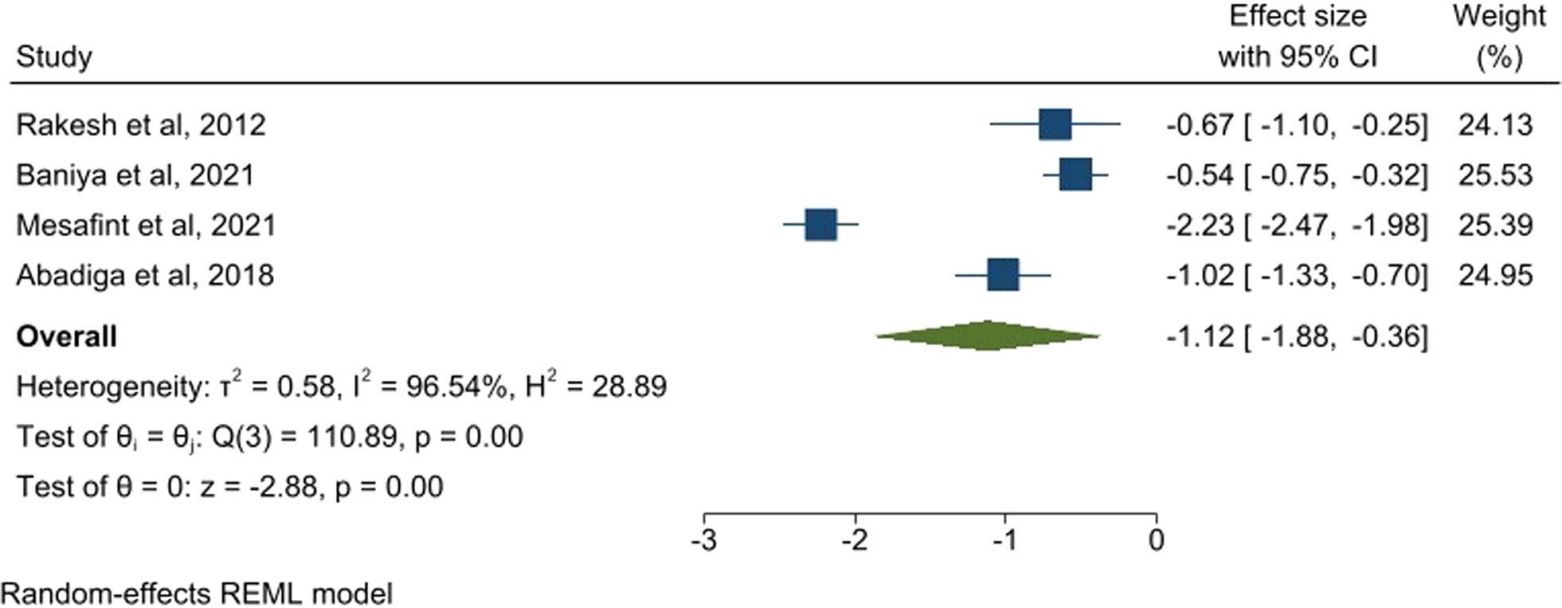
Forest plot of all the studies reporting quality of life (WHOQOL-BREF) in association of comorbid depression

Those studies that utilised WHOQOL-BREF for assessment of quality of life were pooled together and also found a large negative standardized effect size (ES =  − 1.12, (95% CI − 1.88, (− 0.36)), I^2^ = 96.5%) (see Fig. 3).

Tegene et al. [39] also found higher odds of poor QOL in those diagnosed with depression compared to people with epilepsy with no depression (crude OR = 22.39 (95% CI 12.44, 40.30)). Ayanda et al. [40] also found a significant association between comorbid mental health conditions and total score of the WHOQOL questionnaire (p value = 0.044).

A study from Kenya which utilised MOS-36 to assess quality of life found significantly lower total quality of life scores of people with epilepsy with comorbid depression compared to those who were non-depressed (mean QOL score (SD) 46.4 (13.3) versus 64.2 (17.7) [37].

### Comorbid anxiety disorders and quality of life

**Fig. 4:**
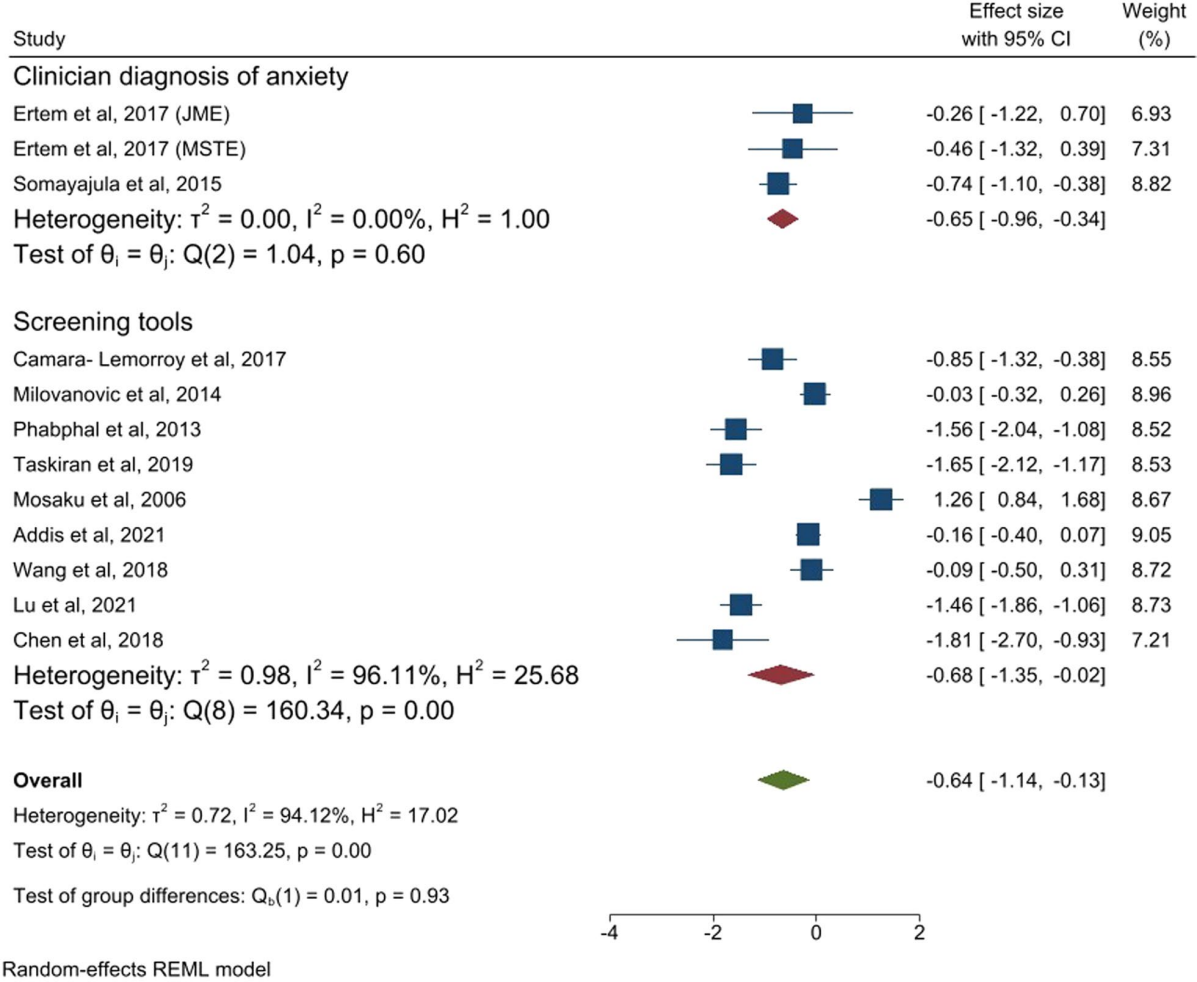
Forest plot of studies reporting quality of life (QOLIE) in association with comorbid anxiety. *MSTE* mesial temporal lobe epilepsy, *JME* Juvenile temporal lobe epilepsy

A total of sixteen studies reported on the association between comorbid anxiety and quality of life [22, 23, 26–30, 32, 36, 39, 45–47, 50, 51, 55]. When all studies using the same kind of instrument for measuring quality of life (QOLIE) were pooled together (eleven out of sixteen) in the meta-analysis, the standard mean effect size was intermediate [pooled ES = −0.64, 95% CI − 1.14, (− 0.13)] (see Fig. 4). There was high heterogeneity across the studies (I^2^ = 94.1%). The median ES (IQR) was − 0.60 (− 1.51, (− 0.13)). The pooled ES was similar for studies which used clinician-based diagnosis and screening tools to measure anxiety.

**Fig. 5:**
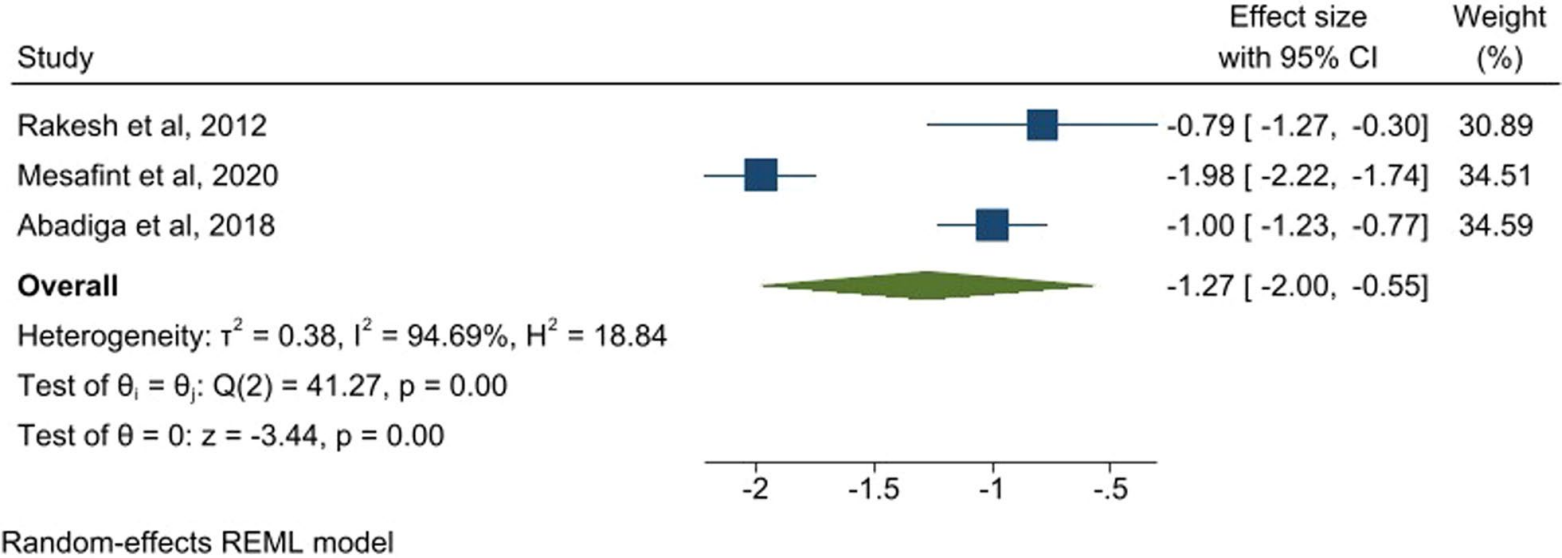
Forest plot of studies reporting quality of life (using WHOQOL-BREF) in association with comorbid anxiety

When the three studies which used WHO-QOL-BREF for measurement of quality of life were pooled together, a large negative mean standardized effect size was found (ES = −1.27, 95% CI − 2.00, (− 0.55); However, there was high heterogeneity (see Fig. 5). Tegene et al., 2015 [39] found those with anxiety had poor quality of life compared to those people without anxiety (crude OR = 9.88 (95% CI 6.05, 16.13).

The number of participants diagnosed with comorbid schizophrenia/psychosis ranged from 1 to 5 and there were no separate analysis reported on its association with quality of life [25, 36, 47].

Most studies adjusted for clinical and socio-demographic factors when assessing the association between comorbid mental health conditions and quality of life except seven studies [31, 36, 43, 47, 48, 51, 55]. The most common confounding factors considered in the multivariable analysis were education and occupation [22, 23, 27, 32, 46, 53], marital status [23–25, 27, 32, 40, 41, 46, 52], seizure frequency [23, 25, 26, 30, 32, 41, 42, 44–46, 49, 50, 52, 56] and number of anti-seizure medications) [23, 24, 27, 28, 30, 32, 49, 52–54]. Some studies also adjusted for age [22–25, 41, 42, 46, 49, 50, 56], sex [22–25, 30, 32, 49, 53] and anxiety symptoms [22–24, 27–29, 32, 45, 46, 50]. All these studies found a significant association between comorbid mental health conditions with quality of life after adjusting except three studies [42, 46, 53]. Sleep disorder and seizure control were included in a multivariable analysis for one study which found no significant association between comorbid depression and quality of life [53]. Another study adjusted for surgery in addition to other variables and depression was no longer significantly associated with quality of life [42].

The association between anxiety symptoms and quality of life was significant in many of the studies which adjusted for multiple socio-demographic and clinical factors except three studies: a study from Mexico (OR = 1.03, 95% CI 0.97 1.09) [28], from Serbia (ß coefficient = 0.188, t = 1.655, p = 0.104) [22] and from Nigeria (ß coefficient = 0.34, p = 0.77) [32].

### Other outcomes

Only one study evaluated the association between comorbid mental health conditions and functioning and found a negative association (multiplier of WHODAS-2 score = 1.83; 95% Confidence Interval (CI) 1.21, 2.76) [25]. Only one study assessed seizure control in relation to comorbid depression and found that co-morbid depression was correlated with seizure control (Pearson Correlation coefficient = 0.37, p = 0.001) [38].

### Quality assessment and risk of bias

The overall assessment of quality of the studies and the risk of bias using AXIS was moderate to low level. The details of scoring and grading of each reviewed study is presented in Additional file 4. There was incompatibility between objectives and study design in four studies [28, 35, 42, 55]. The sample size was not justified in all except nine studies [23–27, 37–39, 41]. The target population was not clearly defined in more than half of the included studies (20 out of 33) and the representativeness of the sample in relation to the reference population was ambiguous in 60% (21 out of 33 studies) of the studies. The sampling strategy, use of unbiased selection of participants, calculation of the sample size and the rate of non-response were not reported in twenty out of thirty three of the reviewed studies [28–32, 35, 36, 38, 40–43, 45, 47–50, 53–55]. Nine of the studies had a sample size less than 100 [28, 29, 32, 36, 37,40, 43, 45, 46]. Both the methods of diagnosing epilepsy and the types of epilepsy were not reported in eight of the reviewed studies [23, 27, 39, 40, 42, 43, 45, 51].

#### Publication bias

**Fig. 6:**
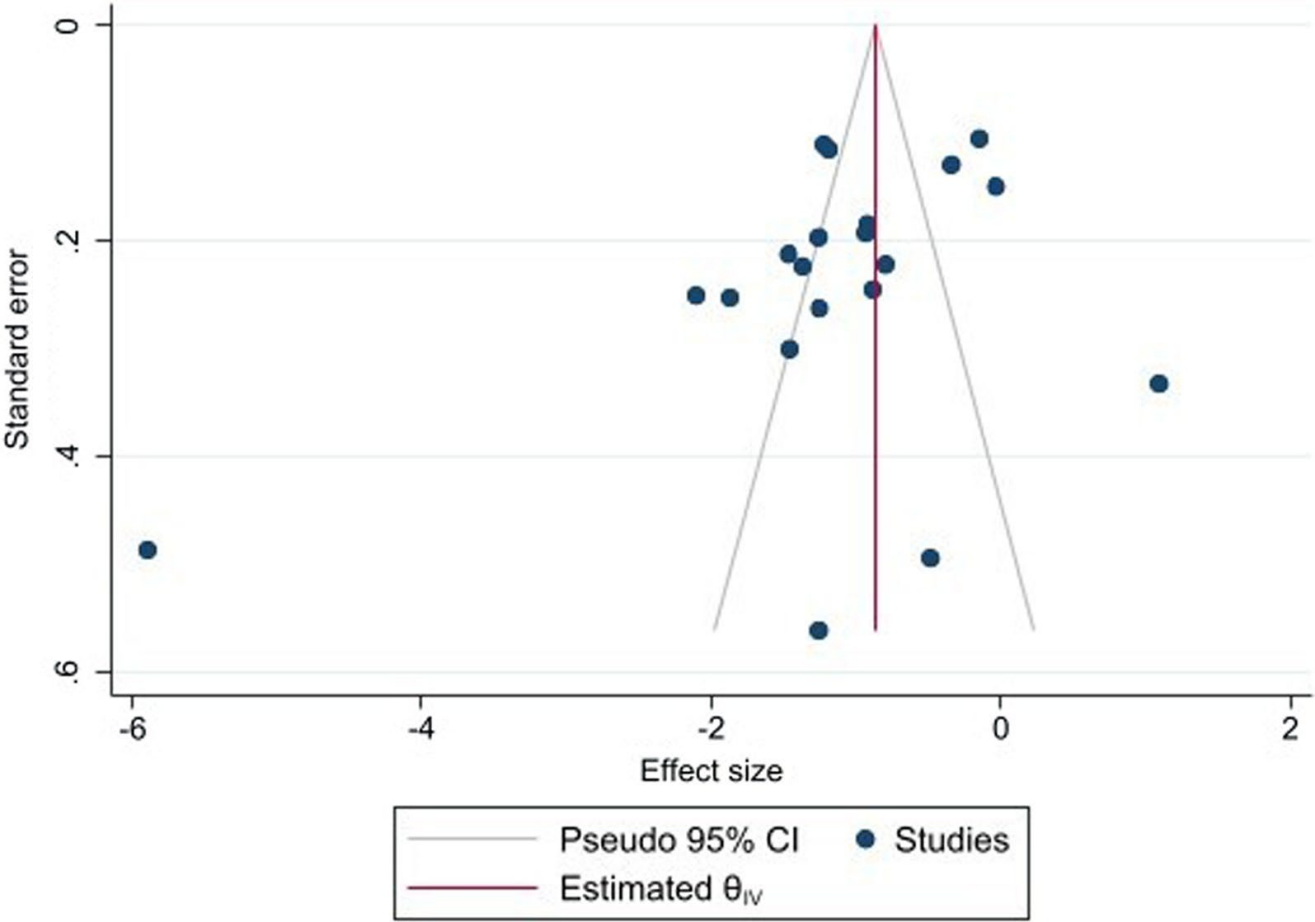
Funnel plot of studies reporting the association of depression with quality of life

Visual inspection of the funnel plot of the nineteen studies included in the meta-analysis for depression and quality of life showed symmetry and there was no evidence of publication bias on the formal test (Begg’s adjusted rank correlation test, p = 0.50). The studies which were scattered outside the funnel plot with large standard errors may show that that there is high variation in the participants of the studies (Fig. 6).

## Discussion

In this systematic review and meta-analysis, we included studies which examined the association of comorbid mental health conditions in people with epilepsy with quality of life or functioning in LMICs. Depression was the most common mental health condition and it showed association with quality of life in the investigated studies. Even though there was high heterogeneity among the studies, the meta-analysis indicated that depression has a large negative effect on quality of life. There was also an intermediate negative effect of anxiety symptoms on quality of life.

This systematic review replicated findings from HIC of strong impacts of depression on the quality of life of people with epilepsy [10, 59]. Regardless of the income status of the country or the type of epilepsy under consideration, the influence of comorbid depression on quality of life was consistently observed. The evidence from HICs is stronger as the association has been measured prospectively [60] and synthesized in systematic reviews [10, 59], whereas we were only able to identify cross-sectional studies for our review. This may overstate the strength of the association and does not illuminate the temporal relationship between co-morbidity and quality of life in people with epilepsy in LMICs. The intermediate effect of anxiety on quality of life was seen but a larger effect was found when the instrument for assessment of quality of life was WHOQOL-BREF. There were far fewer studies which used this generic quality of life assessment tool compared to the epilepsy-specific QOLIE. The intermediate effect of anxiety on quality of life could be due to the higher prevalence of mood disorders than anxiety [4]. Depression has been also observed to be consistently associated with quality of life regardless of the people with epilepsy were treated or refractory to anti-seizure medications s [10].

The findings from this meta-analysis should be interpreted with some caution since most of the analysed studies were found to be moderate to low quality. The study designs of all reviewed studies were cross-sectional, and it is possible findings more reliable designs eg prospective studies may have been different. Cross-sectional studies have the limitation of not identifying the temporal association of comorbid mental health conditions and quality of life or functioning. Almost all the studies recruited participants from outpatient departments of tertiary health care facilities, except four studies [25, 37, 46, 51]. This method of recruitment is likely to be unrepresentativeness of the population of people with epilepsy since most people who are treated at these centres have a more severe form of illness and/or have resources to access these centres. The quality of the included studies was also affected by the lack of justification for the sample size. The overall poor quality and the variation in the methodology of the reviewed studies may have contributed to the statistically high value of heterogeneity. Though there was no difference in the effect size for those studies using the clinician versus screening tools for depression or anxiety, the high heterogeneity of the studies could also be due to the variation in the screening instruments used to measure depression and differences in the application of validated cut off scores between studies. The heterogeneity was slightly lower for those studies which used clinician-based diagnosis (82.5%) of comorbid depression than for those which used screening tools (98.6%), but it was still substantial. This variation could be explained by the difference in the clinical characteristics of the participants (types or duration of epilepsy or the seizure frequency or the different prescribing patterns for anti-seizure medication) and the different eligibility criteria of the studies. The presentation of the results also differed among the reviewed studies which limited the opportunity to pool findings from all the included studies. Potential confounders included within multivariable analyses were inconsistent across the studies which precluded conducting the meta-analysis on the adjusted coefficient. In addition some of the variables like seizure frequency and anti-seizure medications, which may lie on the causal pathway, were considered as confounders. Nonetheless, the findings were still consistent in the adjusted estimates from the individual studies.

Even though there are epilepsy specific screening tools for depression (e.g. neurological disorders depression inventory for epilepsy (NDDI-E)), which has been validated in various languages, the use of that instrument was not seen in most of the reviewed studies [61]. Depression in people with epilepsy could potentially be more accurately identified by using an epilepsy specific rather than a general population depression screening tool [62]. Some of the side effects of anti-seizure medications like sleep disturbance and fatigue could easily be mistaken as the somatic symptoms of depression [62, 63]. NDDI-E was designed to identify depression in people with epilepsy differentiating it from the common side effects of anti-seizure medications [62]. Since most of the reviewed studies have used general population depression screening tools, this may have inflated the diagnosis of the comorbid mental conditions and increase false positivity.

None of the reviewed studies investigated the routine clinical screening and detection of comorbid depression and the impacts of management of the comorbidity on quality of life or functioning. These kinds of research findings could have much more practical and influential evidence to clinicians and policy makers.

We only identified one study which evaluated the impacts of comorbid mental health conditions on functioning of people with epilepsy. As quality of life is considered as a major outcome measurement for health and related services, it could be the reason for high number of studies reporting and measuring it [64]. Though the similar domains (physical, psychological, social and spiritual) are measured in both quality of life and functioning, the accurate measurement of functional status should be objective rather than subjective well-being of the domains [65]. Assessment of functioning for people living in low-income settings has also important implications for survival of the affected individual and for monitoring of outcome of treatment. It is also more reliable measurement of disability associated with epilepsy and comorbid mental health conditions than quality of life.

### Strengths and limitations

This review was comprehensive: we sought to include all eligible studies without any language restriction and covering the main databases with no restriction on date. But we only used English language terms to search the key domains and only English language databases. The main limitation of this review was the lack of high-quality studies which implies the need for future quality studies and the substantial heterogeneity of the studies. There were also a few studies reporting on comorbid psychosis/schizophrenia which indicate the paucity of research on this area.

### Implications

From this systematic review and meta-analysis, it is clear that the effect of depression on quality of life is substantial. The literature from the HICs has recognised this important fact for the last decade and has moved to increasing awareness of the need for early detection and strategies for effective management of depression and anxiety in people with epilepsy [66, 67]. Interventions just targeting seizure control in people with epilepsy are not, therefore, sufficiently comprehensive. Early detection and management of comorbid mental conditions like depression and anxiety could have an important impact on the lives of people with epilepsy, whether they are located in a low- or high-income country. The WHO initiative Mental Health Gap Action Programme (mhGAP) which provides and implements identification and management of prioritized mental health and neurological conditions in LMICS could achieve better treatment outcomes if used in an integrated approach for people with epilepsy and depression. As task-shared psychological interventions for depression have been shown to be effective and feasible in LMICs [68, 69], future intervention trials evaluating the effect of psychological interventions integrated with pharmacological treatments for people with epilepsy are recommended. Future research should also focus on prospective studies which can measure the change in quality of life across time and which can identify the temporal relation between depression and functional disability.

In conclusion the negative effect of comorbid depression and anxiety on quality of life of people with epilepsy living in LMIC is high and comprehensive care mandates early detection and management of these comorbidities in addition to seizure control.

## Supplementary Information

**Additional file 1:** Search terms for Pubmed and other databases.

**Additional file 2:** Data extraction tool.

**Additional file 3:** Sub-group analysis.

**Additional file 4:** Appraisal of Cross-sectional Studies (AXIS).

## Data Availability

All the data generated or analysed during this review are available with this published article as an additional file – data set.

## Abbreviations

ADHD: Attention deficit hyperactivity disorder
AXIS: Appraisal tool for cross sectional studies
BAI: Beck anxiety inventory
BDI: Beck Depression Inventory
CCEI: The Crown Crisp Experiential Index
CI: Confidence interval
CINAHL: Cummulative Index to Nursing and Allied Health Literature
DALYs: Disability adjusted life-years
DSM: Diagnostic and Statistical Manual of mental disorders
EEG: Electroencephalogram
ES: Effect size
GHQ: General Health Questionnaire
GAD-7: Generalized anxiety disorder
GID: Global Index medicus
HADS: Hospital Anxiety and Depression scale
HAMD: Hamilton depression rating scale
HICs: High income countries
ICD: International Classification of Psychiatric Disorders
ILAE: International League Against Epilepsy
IRQ: Interquartile range
JME: Juvenile myoclonic epilepsy
MDI: Major Depression Inventory
MINI: Mini International neuropsychiatric interview
MNS: Mental neurological and substance use disorders
MOS-36: Short version of SF-36
MTLE: Mesial temporal lobe epilepsy
NDDIE: Neurological Disorders Depression Inventory for epilepsy
NOS: Newcastle–Ottawa Scale
OPCRIT: Operational criteria for research
PHQ: Patient Health Questionnaire
PRISMA: Preferred Reporting Items for Systematic Reviews and Meta-analysis PROSPERO-Prospective Register for Systematic Reviews
QOLIE: Quality Of Life for Epilepsy
SAS: Self-rating Anxiety Scale
SCID: Structured Clinical Interview for DSM IV
SDS: The Self-rating Depression Scale
SPS: Social phobia scale
SMR: Standardized mortality ratio
WHOQOL-BREF: World Health Organization Quality of Life Questionnaire
