# Supplementary material for: Co-morbid mental health conditions in people with epilepsy and association with quality of life in low- and middle-income countries: a systematic review and meta-analysis
Source: Health Qual Life Outcomes. 2023 Jan 20;21:5. doi: 10.1186/s12955-022-02086-7 (PMC9854052; doi:10.1186/s12955-022-02086-7)
Supplement: Supplementary file 1 — Additional file 1: Search terms for Pubmed and other databases. [file 12955_2022_2086_MOESM1_ESM.docx]

Search terms for PubMed

| **SN** | **All fields** | **Free terms (Text words)** | **MESH words** | **Result** |
| --- | --- | --- | --- | --- |
| 1 | Epilepsy | “Seizure disorders” OR seizure OR convuls* OR “convulsive disorder” | Epilepsy | 165970 |
| 2 | “Mental disorder” | “psychiatric disorders” OR “common mental disorders” OR “mental distress” OR depression OR “depressive disorders” OR “affective disorders” OR “anxiety disorders” OR phobia OR “panic disorder” OR “social anxiety disorder” OR “somatic symptom disorder” OR “somatoform disorder” OR “illness anxiety” OR, Hypochondriasis OR psychosis OR “psychotic disorders” OR schizophrenia OR schizoaffective OR “delusional disorder” OR “brief psychotic disorder” OR schizophreniform | Disorder, Mental | 698031 |
| 3 | Quality of life | Satisfaction | Quality of life | 661, 781 |
| 4 | LAMIC | Africa OR “Sub-Saharan Africa” OR “low income country” OR “middle income country” OR Afghanistan OR Albania OR Algeria OR “American Samoa” OR Angola OR Armenia OR Azerbaijan OR Bangladesh OR Belarus OR Belize OR Benin OR Bhutan OR Bolivia OR “Bosnia and Herzegovina” OR Botswana OR Brazil OR Bulgaria OR “Burkina Faso” OR Burundi OR “Cabo Verde” OR Cambodia OR Cameroon OR “Central African Republic” OR Chad OR China OR Colombia OR Comoros OR “Democratic Republic of the Congo” OR “Republic of the Congo” OR “Costa Rica” OR “Côte d'Ivoire” OR Cuba OR Djibouti OR Dominica OR “Dominican Republic” OR Ecuador OR Egypt OR “El Salvador” OR Eritrea OR Ethiopia OR Fiji OR Gabon OR Gambia OR Georgia OR Ghana OR Grenada OR Guatemala OR Guinea OR “Guinea-Bissau” OR Guyana OR Haiti OR Honduras OR India OR Indonesia OR Iran OR Iraq OR Jamaica OR Jordan OR Kazakhstan OR Kenya OR Kiribati OR “North Korea” OR Kosovo OR “Kyrgyz Republic” OR "Lao PDR" OR Lebanon OR Lesotho OR Liberia OR Libya OR “North Macedonia” OR Madagascar OR Malawi OR Malaysia OR Maldives OR Mali OR “Marshall Islands” OR Mauritania OR Mauritius OR Mexico OR “Federated states of Micronesia" OR Moldova OR Mongolia OR Montenegro OR Morocco OR Mozambique OR Myanmar OR Namibia OR Nepal OR Nicaragua OR Niger OR Nigeria OR Pakistan OR Palau OR Panama OR “Papua New Guinea” OR Paraguay OR Peru OR Philippines OR Romania OR Rwanda OR Samoa OR “São Tomé and Principe” OR Senegal OR Serbia OR “Sierra Leone” OR “Solomon Islands” OR Somalia OR “South Africa” OR “South Sudan” OR “Sri Lanka” OR “St. Lucia” OR “St. Vincent and the Grenadines” OR Sudan OR Suriname OR Swaziland OR “Syrian Arab Republic” OR Tajikistan OR Tanzania OR Thailand OR Timor-Leste OR Togo OR Tonga OR Tunisia OR Turkey OR Turkmenistan OR Tuvalu OR Uganda OR Ukraine OR Uzbekistan OR Vanuatu OR Vietnam OR “West Bank and Gaza” OR Yemen OR Zambia OR Zimbabwe |  | 5053627 |
| #1 AND #2 | | | | 38050 |
| #1 AND #2 AND #3 | | | | 1,093 |
| #1 AND #2 AND #3 AND #4 | | | | **367** |
| 5 | Functional disability | “activities of daily living” OR rehabilitat* OR capability OR capacity OR work* OR employment OR relationship OR function* | Disabled person | 7,953,263 |
| #1 AND #2 AND #5 | | | | 11,586 |
| #1 AND #2 AND #4 AND # 5 | | | | **42** |
| 6 | “seizure control” | “seizure control” |  | 5432 |
| #1 AND #2 AND # 6 | | | | 867 |
| #1 AND #2 AND #3 AND #6 | | | | **160** |

Search terms for EMBASE

| **SN** | **Key words** |  | | |  | **Result** |
| --- | --- | --- | --- | --- | --- | --- |
| 1 | Epilepsy | | | | |  |
|  | exp reflex epilepsy/ or exp focal epilepsy/ or exp epilepsy/ or exp frontal lobe epilepsy/ or exp catamenial epilepsy/ or exp intractable epilepsy/ or exp grand mal epilepsy/ or exp rolandic epilepsy/ or exp myoclonic astatic epilepsy/ or exp symptomatic epilepsy/ or exp generalized epilepsy/ or exp mesial temporal lobe epilepsy/ or exp "seizure, epilepsy and convulsion"/ or exp lateral temporal lobe epilepsy/ or exp myoclonus epilepsy/ or exp temporal lobe epilepsy/ | | | | | 434268 |
| 2 | Mental |  | | |  |  |
|  | (exp mental disease/ or exp mental stress/ or exp mental health service/ or mental.mp. or exp mental health/ | | | | | 2961200 |
| 3 | LAMIC | Africa OR Sub-Saharan Africa OR low income country OR middle income country | | |  |  |
|  | ((low and middle income country) or Africa or Sub-Saharan Africa or low income country or middle income country).mp. [mp=title, abstract, heading word, drug trade name, original title, device manufacturer, drug manufacturer, device trade name, keyword, floating subheading word, candidate term word] | | | | | 240149 |
| #1 AND #2 |  | | | | | 128822 |
| 4 | **Quality of life** | | Satisfaction | Health related quality of life | |  |
|  | quality of life or satisfaction or health related quality of life).mp. [mp=title, abstract, heading word, drug trade name, original title, device manufacturer, drug manufacturer, device trade name, keyword heading word, floating subheading word, candidate term word] | | | | | 960285 |
| #1 AND #2 AND # 4 |  | | | | | 6870 |
| #1 AND #2 AND #3 AND # 4 | | | | | | **56** |
| 5 | disability | activities of daily living OR rehabilitation OR capability OR capacity OR work OR employment OR relationship OR function | | |  | 808844 |
|  | (disability or activities of daily living or rehabilitation or capability or capacity or work or employment or relationship or function).mp. [mp=title, abstract, heading word, drug trade name, original title, device manufacturer, drug manufacturer, device trade name, keyword heading word, floating subheading word, candidate term word] | | | | | 7872790 |
| #1AND #2 AND #5 |  | | | | | 27845 |
|  | #1AND #2 AND #3 AND #5 | | | | | **149** |
| 6 | Seizure control |  | | |  |  |
|  | seizure control.mp. | | | | | 8846 |
| #1 AND #2 AND #6 |  | | | | | 2327 |
|  | #1 AND #2 AND #3 AND #6 | | | | | **4** |

Search terms for PsycINFO

| **SN** | **Key words** |  | | |  | **Result** |
| --- | --- | --- | --- | --- | --- | --- |
| 1 | Epilepsy  OR Seizure disorders OR seizure OR convulsion OR convulsive disorder | | | | |  |
|  | (epilepsy or Seizure disorder or seizure or convulsion or convulsive disorder).mp. [mp=title, abstract, heading word, drug trade name, original title, device manufacturer, drug manufacturer, device trade name, keyword, floating subheading word, candidate term word] | | | | | 49089 |
| 2 | Mental disorder | psychiatric disorders OR common mental disorders OR mental distress OR depression OR depressive disorders OR affective disorders OR anxiety disorders OR phobia OR panic disorder OR social anxiety disorder OR somatic symptom disorder OR somatoform disorder OR illness anxiety OR, Hypochondriasis OR psychosis OR psychotic disorders OR schizophrenia OR schizoaffective OR delusional disorder OR brief psychotic disorder OR schizophreniform | | |  | 677744 |
|  | (Mental disorder or psychiatric disorders or common mental disorders or mental distress or depression or depressive disorders or affective disorders or anxiety disorders or phobia or panic disorder or social anxiety disorder or somatic symptom disorder or somatoform disorder or illness anxiety OR, Hypochondriasis or psychosis or psychotic disorders or schizophrenia or schizoaffective or delusional disorder or brief psychotic disorder or schizophreniform).mp. [mp=title, abstract, heading word, drug trade name, original title, device manufacturer, drug manufacturer, device trade name, keyword, floating subheading word, candidate term word] | | | | |  |
| 3 | LAMIC | Africa OR Sub-Saharan Africa” OR “low income country OR middle income country | | |  |  |
|  | ((low and middle income country) or Africa or Sub-Saharan Africa or low income country or middle income country).mp. [mp=title, abstract, heading word, drug trade name, original title, device manufacturer, drug manufacturer, device trade name, keyword, floating subheading word, candidate term word] | | | | | 27731 |
| #1 AND #2 | | | | | | 9940 |
| 4 | **Quality of life** | | satisfaction | Health related quality of life | | 230326 |
| #1 AND #2 AND #4 | | | | | | 951 |
| #1 AND #2 AND #3 AND #4 | | | | | | **3** |
| 5 | disability | activities of daily living OR rehabilitation OR capability OR capacity OR work OR employment OR relationship OR function | | |  | 1571430 |
|  | disability or activities of daily living or rehabilitation or capability or capacity or work or employment or relationship or function).mp. [mp=title, abstract, heading word, table of contents, key concepts, original title, tests & measures, mesh word] | | | | |  |
| #1AND #2 AND #5 | | | | | | 2900 |
| #1AND #2 AND #3 AND #5 | | | | | | **21** |
| #1AND #2 AND#3 AND #5 |  | | | | | 3 |
| 6 | Seizure control |  | | |  | 1556 |
| #1 AND #2 AND #6 | | | | | | 276 |
| #1 AND #2 AND #3 AND #6 | | | | | | **0** |

Search terms for CINHAL

| **SN** | **Title** | **Free terms (Text words)** | | | **Word in major subject heading** | | | **Result** |
| --- | --- | --- | --- | --- | --- | --- | --- | --- |
| 1 | Epilepsy | epilepsy or seizures or epileptic | | | seizures or epilepsy or seizure activity or seizure disorder | | | 42212 |
| 2 | Mental disorders | mental health or mental illness or mental disorder or psychiatric illness | | | mental health or mental illness or mental disorder or psychiatric illness | | | 308471 |
| 3 | low and middle income countries or developing countries | low-income or poverty or low socioeconomic status | | | Low and middle income | | | 64057 |
| #1 AND #2 | | | | | | | | 2302 |
| 4 | Quality of life | | quality of life or well being or well-being or health-related quality of life | | | | quality of life or well being or well-being or health-related quality of life | 291, 454 |
| #1AND #2AND #4 | | | | | | | | 271 |
| #1AND #2AND #3AND #4 | | | | | | | | **22** |
| 6 | Disability | | function | | | | Disability | 411836 |
| #1 AND #2 AND #6 | | | | | | | | 411 |
| #1 AND #2 AND #3AND #6 | | | | | | | | **21** |
| 7 | Seizure control | | | Seizure control | | Seizure control | | 2143 |
| #1 AND #2 AND #7 | | | | | | | | 88 |
| #1 AND #2 AND #3AND #7 | | | | | | | | **2** |

Search terms for GIM

| **SN** | **Title , abstract and subject** | | | **Result** |
| --- | --- | --- | --- | --- |
| 1 | Epilepsy | “Seizure disorders” OR seizure OR convuls* OR “convulsive disorder” |  | 122,069 |
| 2 | “Mental disorder” | “psychiatric disorders” OR “common mental disorders” |  | 27674 |
| 3 | Quality of life | Health related quality of life |  | 23573 |
| #1 AND #2 |  | | | 24,129 |
| #1AND #2 AND #3 |  | | | **960** |
| 5 | Disability | Functional disability OR activities of daily life |  | 9397 |
| #1AND #2 AND #5 |  | | | **286** |
| 6 | Seizure control |  |  | 1975 |
| #1 AND #2 AND #6 |  | | | 0 |
