# Supplementary material for: Co-morbid mental health conditions in people with epilepsy and association with quality of life in low- and middle-income countries: a systematic review and meta-analysis
Source: Health Qual Life Outcomes. 2023 Jan 20;21:5. doi: 10.1186/s12955-022-02086-7 (PMC9854052; doi:10.1186/s12955-022-02086-7)
Supplement: Supplementary file 2 — Additional file 2: Data extraction tool. [file 12955_2022_2086_MOESM2_ESM.docx]

**Data extraction tool**

| Study title or ID |  |
| --- | --- |
| Study ID *(surname of first author and year first full report of study was published e.g. Smith 2001)* |  |
| Report ID |  |
| Report ID of other reports of this study including errata or retractions |  |
| Notes | |

# General Information

| Date form completed *(dd/mm/yyyy)* |  |
| --- | --- |
| Name/ID of person extracting data |  |
| Reference citation |  |
| Study author contact details |  |
| Publication type *(e.g. full report, abstract, letter)* |  |
| Notes: | |

# Study eligibility

| Study Characteristics | Eligibility criteria  *(Insert inclusion criteria for each characteristic as defined in the Protocol)* | | Eligibility criteria met? | | | Location in text or source *(pg & ¶/fig/table/other)* |
| --- | --- | --- | --- | --- | --- | --- |
|  |  | | Yes | No | Unclear |  |
| Type of study | Cross- sectional | |  |  |  |  |
|  | Case control | |  |  |  |  |
|  | Cohort  Prospective  Retrospective | |  |  |  |  |
|  | *Validation studies* | |  |  |  |  |
|  | Other design (specify): | |  |  |  |  |
| Participants  18 – 65 years |  | |  |  |  |  |
| Types of the tool / instrument used for assessment |  | |  |  |  |  |
| Is the instrument validated |  | |  |  |  |  |
| Types of outcome measures |  | |  |  |  |  |
| INCLUDE | | EXCLUDE | | | | |
| Reason for exclusion |  | | | | | |
| Notes: | | | | | | |

# Characteristics of included studies

## Methods

|  | **Descriptions as stated in report/paper** | | **Location in text or source** *(pg & ¶/fig/table/other)* |
| --- | --- | --- | --- |
| **Aim of study** |  | |  |
| **Design** |  | |  |
| **Country & category of income** |  | |  |
| **Setting ( 1, 2 or 3 centres)** |  | |  |
| **End date** |  | |  |
| **Duration of follow up for the cohort studies** |  | |  |
| **Ethical approval needed/ obtained for study** | Yes No Unclear |  |  |
| **Notes:** | | | |

## Participants

|  | Description | | Location in text or source *(pg & ¶/fig/table/other)* |
| --- | --- | --- | --- |
| Population description *(from which study participants are drawn)* |  | |  |
| Setting *(including location and social context)* |  | |  |
| Inclusion criteria |  | |  |
| Exclusion criteria |  | |  |
| Method of recruitment of participants *(e.g. phone, mail, clinic patients)* |  | |  |
| Informed consent obtained | Yes No Unclear |  |  |
| Total sample size ( cases ) |  | |  |
| Clusters *(if applicable, no., type, no. people per cluster)* |  | |  |
| Baseline imbalances for case control studies |  | |  |
| Withdrawals and exclusions *(if not provided below by outcome)* |  | |  |
| Age |  | |  |
| Sex |  | |  |
| Race/Ethnicity |  | |  |
| Types of epilepsy |  | |  |
| How is the diagnosis of epilepsy done   - Clinician - EEG based - Others |  | |  |
| Co-morbidities |  | |  |
| Other relevant socio-demographics |  | |  |

## Exposure status (comorbidities of mental disorders)

|  | Description as stated in report/paper | Location in text or source *(pg & ¶/fig/table/other)* |
| --- | --- | --- |
| Diagnosis of the comorbidity |  |  |
| *Proportion of people with the comorbidity* |  |  |
| How is the detection of comorbidity of CMD done   - Screening using a tool - Clinician - other |  |  |
| Validation of the CMD assessment tool |  |  |
| Notes: | | |

## Outcomes

*Copy and paste table for each outcome.*

**Outcome 1**

|  | Description as stated in report/paper | | Location in text or source *(pg & ¶/fig/table/other)* |
| --- | --- | --- | --- |
| Outcome name |  | |  |
| Time points measured ( follow up period ) |  | |  |
| Outcome definition *(with diagnostic criteria if relevant)* |  | |  |
| Person measuring/ reporting |  | |  |
| Unit of measurement *(if relevant)* |  | |  |
| Scales: upper and lower limits *(indicate whether high or low score is good)* |  | |  |
| Is outcome/tool validated? | Yes No Unclear |  |  |
| Imputation of missing data |  | |  |
| Assumed risk estimate *(e.g. baseline or population risk noted in Background)* |  | |  |
| Power *(e.g. power & sample size calculation, level of power achieved)* |  | |  |
| Notes: | | | |

## Other

| **Study funding sources** *(including role of funders)* |  |  |
| --- | --- | --- |
| **Possible conflicts of interest** *(for study authors)* |  |  |
| **Notes:** | | |

**Main findings**
