# Supplementary material for: Co-morbid mental health conditions in people with epilepsy and association with quality of life in low- and middle-income countries: a systematic review and meta-analysis
Source: Health Qual Life Outcomes. 2023 Jan 20;21:5. doi: 10.1186/s12955-022-02086-7 (PMC9854052; doi:10.1186/s12955-022-02086-7)
Supplement: Supplementary file 3 — Additional file 3: Sub-group analysis. [file 12955_2022_2086_MOESM3_ESM.docx]

**Sub- group analysis**


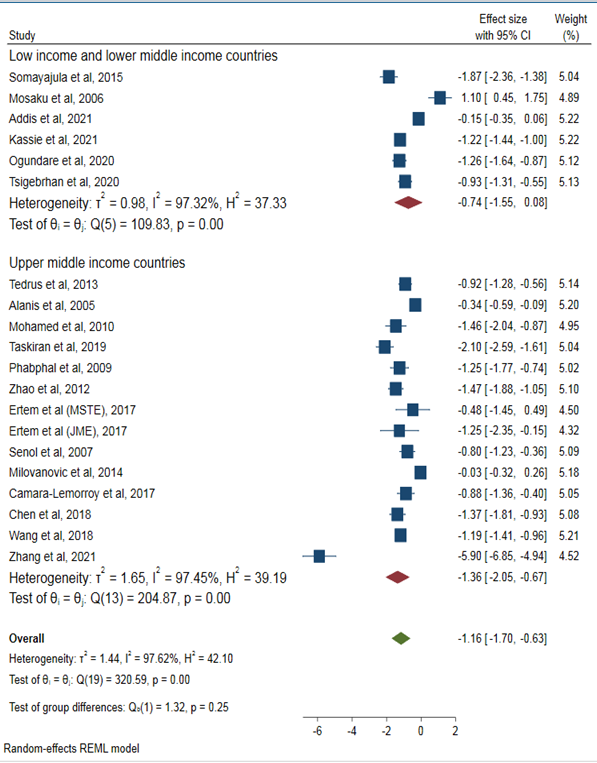


Figure 1. Forest plot of all the studies reporting the quality of life (QOLIE) in association of comorbid depression sub- grouped by the income categories of the study countries. MSTE-mesial temporal lobe epilepsy, JME- Juvenile temporal lobe epilepsy


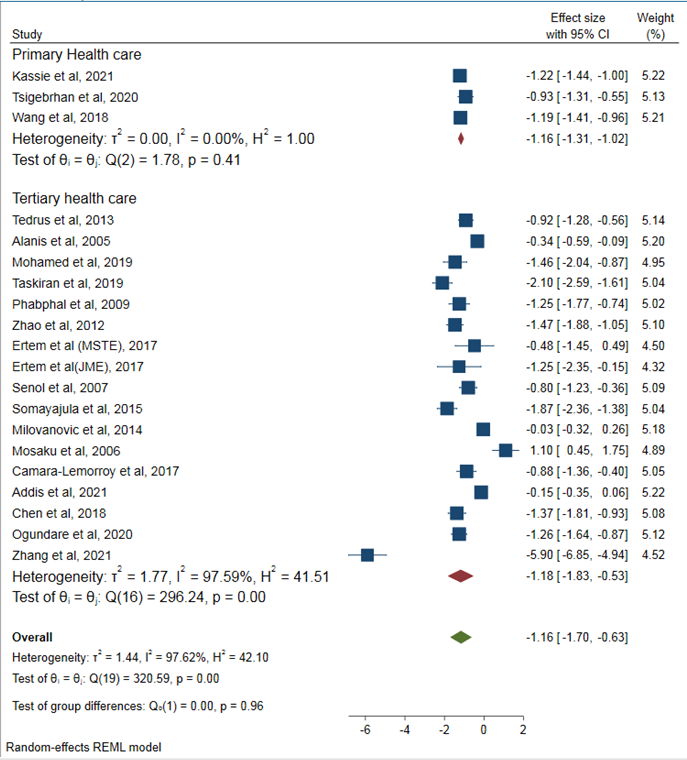


Figure 2.Forest plot of all the studies reporting the quality of life (QOLIE) in association of comorbid depression sub- grouped by setting. MSTE-mesial temporal lobe epilepsy, JME- Juvenile temporal lobe Epilepsy
